# Fetal Neurology: From Prenatal Counseling to Postnatal Follow-Up

**DOI:** 10.3390/diagnostics12123083

**Published:** 2022-12-07

**Authors:** Barbara Scelsa

**Affiliations:** Department of Pediatric Neurology and Psychiatry, V. Buzzi Children’s Hospital, ASST-FBF-Sacco, via Castelvetro 32, 20154 Milan, Italy; barbara.scelsa@asst-fbf-sacco.it

**Keywords:** fetal counseling, fetal neurology, ventriculomegaly, corpus callosum agenesis, posterior fossa malformations, neurodevelopmental outcome

## Abstract

Brain abnormalities detected in fetal life are being increasingly recognized. Child neurologists are often involved in fetal consultations, and specific fetal neurology training has been implemented in many countries. Pediatric neurologists are asked to examine the data available and to contribute to the definition of the long-term outcomes. Ventriculomegaly, posterior fossa malformations, and agenesis/dysgenesis of corpus callosum are among the most common reasons for antenatal neurological consultations. Fetuses with central nervous system and extra-CNS anomalies should ideally be managed in secondary/tertiary hospitals where obstetricians who are experts in fetal medicine and pediatric specialists are available. Obstetricians play a critical role in screening, performing detailed neurosonography, and referring to other specialists for additional investigations. Clinical geneticists are frequently asked to propose diagnostic tests and counsel complex fetal malformations whose phenotypes may differ from those during postnatal life. Advances in fetal MRI and genetic investigations can support the specialists involved in counseling. Nevertheless, data interpretation can be challenging, and it requires a high level of expertise in a multidisciplinary setting. Postnatally, child neurologists should be part of an integrated multidisciplinary follow-up, together with neonatologists and pediatricians. The neurodevelopmental outcomes should be assessed at least up to school age. Children should be evaluated with formal tests of their gross motor, cognitive, language, fine motor/visuo-perceptual skills, and their behavior. In this perspective, fetal neurology can be regarded as the beginning of a long journey which continues with a prolonged, structured follow-up, support to the families, and transition to adult life. A review of the most common conditions is presented, along with the long-term outcomes and a proposal of the neurodevelopmental follow-up of children with CNS malformation which are diagnosed in uterus.

## 1. Introduction

The role of pediatric neurologists during fetal life is now well recognized [1]. However, only recently has specific fetal neurology training been proposed and implemented [2]. Considering the complex and fragile maternal–fetal dyad, a high level of training and experience is necessary. Fetal counseling is a multidisciplinary discipline [3]. It typically cannot be performed by only one specialist, and it requires the presence of a team of experts, including the referring obstetrician, the neuroradiologist, the child neurologist, the geneticist, the psychologist, and other pediatric subspecialists in case of extra-central nervous system (CNS) anomalies. In addition, the specialists involved must be not only experienced, but also empathic and able to communicate the prognosis/diagnosis to the parents. 

Central nervous system abnormalities (CNS) in the fetus can be the result of maternal, fetal, and placental disorders [2]. CNS malformations are the result of the impairment of a complex and orchestrated process at various embryonic or fetal stages. Abnormal neurulation, telencephalic division, neuronal proliferation, migration, and cortical organization may play a role in the pathogenesis of CNS malformations. Abnormalities in one or more of these developmental processes may be caused by specific chromosomal disorders or monogenic mutations. Fetal brain anomalies, often in association with extra-CNS disruptions, can be the result of chromosomal aberrations, as in agenesis of corpus callosum caused by mosaic trisomy 8 and 1q43q44 microdeletion syndrome. Mosaic trisomy 9 and several unbalanced translocations can cause posterior fossa malformations [4]. Monogenic mutations are usually associated with significant neurodevelopmental disabilities, such as agenesis of corpus callosum secondary to mutations of EPG5, ZEB2, SLC12A6, and AIC genes, or in posterior fossa anomalies caused by mutations of OPHN1, FMR1, RPL 10, DKC1, and ZIC3 [5]. However, mutations in the DCC Netrin 1 receptor gene can cause the isolated agenesis of corpus callosum with only minor neurological signs and a normal neurodevelopmental outcome [6]. Embryology should always be the starting point for any specialist involved in fetal counseling in order to recognize not only genetic etiologies, but also trimester-specific mechanisms affecting the fetal development [7]. An injury to the fetal brain may evolve into brain malformations when it occurs in the first trimester or into destructive lesions when it occurs later during gestation [7,8]. Alcohol abuse, malnutrition, vascular insults, and congenital infections can be responsible for several brain malformations when they occur early during gestation (i.e., corpus callosum agenesis in alcohol abuse and polymicrogyria in CMV infections).

This review focuses on how to structure a fetal neurological consultation and how to approach some of the most common CNS abnormalities encountered in fetal life. A section will also be dedicated to how to structure a postnatal follow up.

## 2. The Framework of Fetal Neurological Consultations

### 2.1. Data Collection

Accurate anamnestic information regarding family medical history and consanguinity should be obtained. Pregnancy history, the need for assisted reproductive procreation, the outcome of prior pregnancies, previous testing, including a serological evaluation of TORCH, maternal exposure to toxic substances, and medications are all important information. It is also relevant to know if the parents can count on a family network. Family members can be a support system for parents when they are making decisions or when they are organizing optimal planning for postnatal assistance and the care of an infant who may have multiple disabilities. 

### 2.2. Imaging

A standard fetal ultrasound allows us to observe live fetal structures, growth, amniotic fluid, and to screen for CNS and extra-CNS abnormalities. Neurosonography is a further step that can diagnose complex conditions with advanced technologies when it is performed by an expert obstetrician [9]. Fetal MRI is considered complementary to neurosonography, and it is usually performed when an ultrasound is inconclusive and/or when specific abnormalities are suspected. The rate of associated malformations detected only by MRI is related to the type of ultrasound that is performed. As recommended by the International Society of Ultrasound in Obstetrics and Gynecology (ISUOG), fetuses presenting with CNS anomalies should undergo dedicated neurosonography before referring to fetal MRI [10]. When the fetuses undergo accurate neurosonography, fetal MRI can detect additional abnormalities in 5% of the cases with ventriculomegaly [9,11]. In the fetuses with corpus callosum dysgenesis, an MRI can identify additional structural anomalies in 11.2% of the cases [12]. Undetected anomalies during the ultrasound in conditions such as ventriculomegaly, vermian hypoplasia, and corpus callosum agenesis may significantly change the prognosis of these children, and this may have an impact on the parents’ decisions. The most common brain anomalies that may be diagnosed only by fetal MRI are cortical and posterior fossa malformations. In this respect, fetal MRI should be considered to be an essential tool in adjunction to neurosonography [12,13]. Fetal MRI represents the gold standard for studying the dynamic process of cerebral cortex formation. Righini et al. suggested that the abnormal development of the subplate and the intermediate zone can offer insights and assist in the diagnosis, even of focal cortical malformations before 24 weeks of gestation when the brain has a smooth appearance [14,15]. The cerebral cortex is derived from the telencephalic pallium with an inside-out pattern: the last neurons migrating constitute the most superficial layers. The inner layer is the germinal zone where the neurons proliferate and progressively migrate towards the surface layers: the subplate, the intermediate zone, and the cortical plate. The subplate and the intermediate zone constitute the precursors of the white matter. More specifically, the subplate zone is a transient area in cortical formation: it represents a waiting compartment for cortical afferents [16]. Righini et al. describe four MRI patterns of a cortical plate anomaly which constitute the early signs of cortical malformations: multiple small bulging areas (wart-like areas), abnormal sulcation, multiple small spikes alternating to small invaginations (sawtooth), and abnormal bumps [14]. In addition, abnormal thickness or the blurring of the subplate and intermediate zones detected by MRI can be considered to be an indirect sign of abnormal cortical connections in the developing cortex [14,15]. In case of uncertainty or conditions which may change over time, an additional fetal MRI could be performed after a few weeks if the gestational age permits it. Nevertheless, the parents should also be aware that even after fetal MRI, apparently isolated malformations may be postnatally reclassified as complex conditions.

### 2.3. Genetic Testing

Microarray Comparative Genomic Hybridization (CGH) is now routinely offered in the place of the karyotype. It detects microdeletions and microduplications. More recently, next generation sequencing (NGS) has been used to analyze a large number of genes at the same time. However, the fetal phenotype of monogenic mutations may be different or limited in comparison with the postnatal phenotype. For this reason, NGS should be recommended in selected conditions [17]. Whole exome (WES) or genome (WGS) sequencing is the ultimate frontier [18]. A recent meta-analysis revealed that in CNS malformations, the diagnostic yield of exome sequencing over microarray CGH is 17% [19]. However, in the Population Architecture through Genomics and Environment (PAGE) study, WES detected a genetic variant in 8.5% of the fetuses with isolated brain anomalies, a percentage lower than that which was expected from previous small-size studies [20]. On the contrary, in fetuses with associated extra-CNS involvement, a diagnostic genetic variant was found in 15.4% of the cases [20]. The PAGE study recommends that WES should be limited to pregnancies in which it is most likely to be diagnostic (multi-system malformations) to avoid situations in which the detection of variants of uncertain significance (VUSs) would increase the uncertainty in fetal counseling. At the same time, caution must be taken before offering WES. WES is not only an expensive test, but it also requires a longer processing time, which is not always feasible in countries and contexts where there is a time limit for the legal termination of the pregnancy. Non-invasive prenatal diagnosis (NIPD) can be performed very early during pregnancy, but its utility seems to be limited to screening for common chromosomal anomalies. However, more recently, its use has been proposed to study exome sequencing, but the results are still unreliable [21]. On the contrary, relative haplotype dosage analysis (RHDO) seems to be a promising non-invasive technique to diagnose single gene mutations in the cell-free fetal DNA released into the maternal circulation [22]. A detailed description of fetal genetic investigations is beyond the scope of this study, but a number of excellent reviews are available [17,19,20,21,22,23,24,25].

### 2.4. Multidisciplinary Team 

Before meeting the parents, the specialists involved in the consultation need to review, collegially, all of the information, imaging, and anamnestic data [3]. This moment is essential to tailor the counseling, especially when the CNS abnormalities are minor or subtle and the long-term outcome is uncertain [26]. Some anomalies when they are isolated may be variants of normality, making the parental decisions even more difficult. Moreover, in many countries, the termination of the pregnancy has a time constraint. In Italy, the legal time for termination of pregnancy is around 23 weeks. Taking this into consideration, the investigations must be carried out before that time, and often, a more extensive genetic analysis such as WES cannot support the diagnostic process. The specialists are asked to interpret the results in their field of expertise, and concerted communications should be collegially agreed upon. The fetus phenotype may differ from that described in postnatal life, and each specialist may offer specific contributions. The under- or over-estimation of information can lead to incorrect interpretations. In addition, the parents have to make difficult decisions, and they must have the opportunity to ask questions in person whenever this is possible. Telemedicine can be an alternative when in-person consultation is not feasible. 

### 2.5. Counseling Setting

Antenatal consultations require a formal setting, which must have dedicated time and space. The communication cannot be effective when it is offered while one is standing in a passageway and in a limited time frame. The counseling should be held in a quiet room in the presence of the members of the multidisciplinary team who are directly involved [8]. The obstetrician and the child neurologist should always be available. Additional specialists are summoned when there are extra-CNS malformations (i.e., a cardiologist, a surgeon, an orthopedic, and a pediatrician). Geneticists should be available when genetic conditions are suspected. Telemedicine may be an additional resource when a specialist is unable to be present. During the COVID-19 pandemic, many fetal consultations in our institution were carried out as remote video conferences using our hospital platform (COD20©).

Although the conditions and malformations may be complex, and the outcome may be uncertain, the use of excessive technical and medical terminology should be avoided. Risk factors, percentages, and numbers are certainly practical and should be presented, but the parents also want to know the impact of their child’s condition in real life. The communication of a severe prognosis may be overwhelming, and an excessive amount of data may be confusing and difficult to remember. Anatomical drawings or other iconographic images should be available. The communication of the prognosis and/or diagnosis may be better understood when a visual illustration is presented. However, MRI or ultrasound images of the fetus should be shown only after a specific request. The parents might decide to terminate the pregnancy, where it is allowed, and being exposed to real “pictures” of their child can be extremely painful. On the contrary, when the parents choose to continue the pregnancy despite the presence of a malformation, the maternal bonding to the fetus should be protected, avoiding unnecessary graphic images. The parents must be allowed to ask questions and to have the necessary time to process the information. During counseling, the parents may need a pause in a private space to freely express their sorrow and feelings to one another. The pediatric neurologist and the other specialists should provide empathy and exhibit consideration for the parents’ decisions. Each consultation must be tailored to the individual needs [27]. When one is dealing with unfamiliar cultures and languages, it is advised to seek help from a cultural and language mediator. Their priorities, sets of values, and beliefs may be different. In fetal counseling, it is essential to learn how to communicate in the best and most effective way in specific contexts and cultures. It is important to emphasize that fetal counseling should be carried out in secondary/tertiary care institutions. It is strongly recommended to refer pregnant women with CNS fetal malformations to institutions where there is an experienced fetal medicine unit, fetal MRI is performed routinely, and pediatric specialists are familiar with rare diseases and the postnatal follow-up.

## 3. Fetal CNS Abnormalities

The pediatric neurologist is often asked to comment and counsel the patients regarding anomalies which may be already defined in fetal life and have a specific prognosis. However, unclear and less defined conditions are more frequently encountered, making counseling and the parent’s decisions very difficult. 

In the literature, CNS fetal malformations are usually studied in small and not homogenous groups of patients, often with a short and not standardized follow-up. Considering the rarity of some malformations, a large series of fetuses with an isolated CNS malformation in a single center is difficult to acquire. For this reason, it is not unusual to have studies which include anomalies associated with extra-CNS or other brain disorders under the same designation. The same brain abnormality that occurs in fetal life may be the result of genetic or interfering conditions, namely infections, vascular injuries, or metabolic imbalance. It would be more accurate to study the conditions in groups that are as homogenous as possible. For this reason, fetal consultations should be approached differently compared to those during postnatal life. As already mentioned, the first step is collecting as much information as possible in a short time frame from the imaging, genetic testing, pregnancy, and family history data. The effort of the multidisciplinary team, and especially of the pediatric neurologist, is required to translate all of the information into a possible diagnosis and prognosis. 

This review will consider some of the most frequent and less defined malformations, which are in the gray area, and which are difficult to prognosticate. The conclusions are summarized in Table 1. 

### 3.1. Isolated Ventriculomegaly

Ventriculomegaly (VM) is probably one of the most common reasons for fetal counseling [9]. The incidence of isolated VM is between 0.4 and 0.9 per 1000 births [38]. It is defined as a measurement of more than 10 mm at the level of the ventricular atria. According to the ISUOG guidelines, the atrial measurement remains stable in the second and mid-third trimesters of pregnancy, with a mean diameter that is between 6 and 8 mm [10].

VM is classified as mild (10–12 mm), moderate (13–15 mm), or severe (>15 mm). It is more frequent in males, although its prognosis is not determined by sex. The etiology, the dilation progression, the severity, and the presence of associated anomalies constitute the determining factors in predicting the outcome. Infections, genetic conditions, additional CNS and extra-CNS abnormalities should be ruled out. When VM is not progressive or associated with other CNS and extra-CNS anomalies, it is referred to as isolated, and the prognosis is related to the size of the ventricles [39,40]. An MRI should be included in the prenatal investigations, especially when the ventricles have a moderate or a severe enlargement [11,40]. In a recent multicenter study, fetal MRI detected additional abnormalities in 18% of the fetuses with isolated severe VM on neurosonography, with cortical malformations being the most significant finding [40]. In mild/moderate VM, postnatal MRI detected 3.8% of the additional anomalies that were missed in the fetal images [11]. Nevertheless, the European Neurosonography (ENSO) Working Group suggests performing fetal MRI for every fetus with a prenatal diagnosis of mild/moderate VM, “although parents can be reassured of the low risk of an associated anomaly when VM is isolated on neurosonography” [11]. In addition to the detection of additional malformations, congenital infections and genetic conditions must be ruled out. Microarray CGH is now largely used, and it has replaced karyotype analysis in most centers. According to Toren et al., microarray CGH aberrations are more common in the non-isolated ventriculomegaly cases (24.1%) compared to in the controls (6.2%) [41]. However, the rate of genetic aberrations does not seem to be associated with the degree of dilatation [41]. In a recent retrospective analysis, chromosomal karyotype abnormalities were detected in 1% of the cases with isolated VM, while no pathogenic CNVs were detected [42]. On the contrary, an additional 10% of pathogenic CNVs were found in isolated VM by Chang in a large study [43]. Even though CNVs probably have a lower detection rate than they have been previously described to have, the Society for Maternal-Fetal Medicine recommends amniocentesis with microarray CGH when ventriculomegaly is detected [39].

In truly isolated ventriculomegaly, the neurodevelopmental outcome is favorable in over 90% and 75–93% of children with mild and moderate ventriculomegaly, respectively [27,39]. Nevertheless, a limit of many studies is the use of telephone interviews or clinical reports [27]. In addition, very few authors describe the long-term neurodevelopmental outcome. Most of the studies limit the follow-up to the first three years of life. However, subtle neurodevelopmental impairments may be detectable only later in life. There is only one long-term study with a comprehensive cognitive, motor, and visuo-perceptual follow-up [31]. It includes a small cohort of 17 children with isolated fetal ventriculomegaly (10–15 mm), none of whom were submitted to fetal or postnatal MRI. Only one child with minor motor difficulties was identified [31]. On the other hand, it has been suggested that VM is a marker of abnormal cortical growth, which may have implications in developing psychiatric and other neurodevelopmental disorders [44]. Recently, global and regional changes of cortical development were described in fetuses with isolated mild/moderate ventriculomegaly (10–14.9 mm) [45]. A significant increase in the cortical gray matter volume was found in the posterior part of the cingulate gyrus and the anterior part of the gyri parahippocampalis and ambiens [45]. In addition, decreased cortical volumes were observed in the frontal lobe of both hemispheres. The authors suggested the possibility that VM may be associated with an increased number of or a reduced apoptosis of the progenitor cells. They hypothesized that VM might be an indirect sign of cytoarchitectonic changes, which may affect neuronal interaction and functionality [45]. These findings may be used as a potential biomarker to identify the children at risk of developing neurodevelopmental disabilities [46,47]. Therefore, a long-term follow-up at school age and beyond is necessary to clarify the implication of the cortical changes associated with VM.

### 3.2. Isolated Corpus Callosum Agenesis (cACC)

The formation of the corpus callosum starts at as early as 6 weeks of gestation, and it is formed by 18 weeks and reaches full development in adolescence [48]. Various infections, vascular, toxic, metabolic, and genetic elements can interfere with its development. There is no univocal consensus in the terminology used to describe corpus callosum abnormalities. In this review, we will consider the classification used by Leombroni et al. and Hanna R. et al. [49,50]. Agenesis (ACC) is when the corpus callosum is completely absent (cACC) or partially absent (pACC). Hypoplasia is defined by the presence of a fully formed but thinner corpus callosum. It is not uncommon to describe a corpus callosum with hypoplasia of one of its components (often the posterior part) or partial agenesis. The latter is usually not isolated, and it is the result of late damage in metabolic disorders (phenylketonuria and peroxisomal disorders), infections (toxoplasmosis and CMV), and an ischemic insult. On the other end, hyperplasia is characterized by the presence of a fully formed but thick corpus callosum. Dysplasia refers to a corpus callosum, which does not reach a normal level of differentiation and takes abnormal shapes. The latter is often detected in association with other CNS abnormalities, mainly neuronal migration disorders [49]. In the general population, the incidence of corpus callosum agenesis is 0.020–0.025%, while in the subjects with neurodevelopmental disabilities, it is 1–3% [49]. More than 250 syndromes are associated with cACC. Chromosomal abnormalities are found in 4.8% of subjects with cACC [51]. Prenatal genetic testing may detect abnormal findings in 18% of the cases. For this reason, most authors recommend offering genetic investigations of the fetus, such as microarray CGH and WES [24,51,52]. Nevertheless, it has been suggested that the co-occurrence of genetic and environmental factors plays a critical role in corpus callosum abnormalities [51].

Most of the clinical reports and meta-analyses describe that 61–70% of children prenatally diagnosed with isolated cACC have normal neurodevelopment [30,53,54]. Fetal MRI may detect additional abnormalities, such as cortical malformations, in about 22–40% of the patients [30,55]. Mild (language disorders and dyspraxia) and severe disabilities are found in 14–27% and 8.2–12.5% of them, respectively [30,53]. Severe outcomes are usually associated with genetic conditions, which were not diagnosed in fetal life [54]. Despite the normal cognitive development in most of the children with isolated cACC, subtle deficits can be disclosed later in life [56]. Semantics and developmental language disorders have been described in cACC subjects, such as difficulties in understanding humoristic situations, proverbs, and in verbal fluency [57]. In addition, it has been reported that, when the follow-up is prolonged and standardized, 50% of the children experience some difficulties in executive functions, impairing their scholastic achievement [29,58]. Social functioning can also be affected: people with cACC may have a reduced level of empathy, and an inaccurate self-awareness of their limits and the consequences of their choices. They may function relatively well in highly routinized social interactions, but in more demanding situations they may exhibit symptoms of distress and inhibition [56]. In infancy and at school age, these symptoms may not be perceived. It has been suggested that social and executive functions impairments may be better defined in adolescence, even if the child previously showed normal cognitive and neuropsychological development. In adolescence, the development and myelination of corpus callosum are completed, but other cerebral commissures are unable to compensate for an increasingly demanding environment and the exposure to more complicated situations. For all of these reasons, the follow-up should be continued until adolescence and early adulthood to support them and offer dedicated treatments [59].

### 3.3. Posterior Fossa Malformations

The posterior fossa malformations include abnormalities of the cerebellum, the cerebral peduncles, the fourth ventricle, the pons, the medulla, the cisterna magna, and the tentorium. All of the structures of the posterior fossa are derived from the rhombencephalon. By the fifth/sixth gestational week, the pontine flexure separates the rhombencephalon into the metencephalon and the myelencephalon [60]. In posterior fossa malformations, neurosonography and fetal MRI are complementary to substantiate the diagnosis and rule out any associated anomalies which may modify the prognosis [34]. There is increasing evidence that the cerebellum is not only involved in motor abilities and coordination, but it might be associated with cognitive and behavioral skills [61]. Several studies have reported a correlation between autism spectrum disorders and vermis morphology [62]. For this reason, long-term outcome counseling may be challenging in pregnancies with posterior fossa malformation.

This review will focus only on the more common conditions in which the prognosis may be uncertain.

#### 3.3.1. Blake’s Pouch

During its development, the posterior fossa undergoes numerous changes, including the appearance and disappearance of several structures. One of the most significant ones is Blake’s pouch which disappears at approximately 18 weeks of gestation with the closure of the fourth ventricle. The differential diagnosis of Blake’s pouch with other conditions, such as an arachnoid cyst, mega cisterna magna, cerebellar hypoplasia/vermian hypoplasia, and Dandy–Walker spectrum can be challenging for the prognostic implications [63]. The prognosis of Blake’s pouch tends to be favorable in most cases, however, the same does not always apply to the other conditions. By the third trimester most of the Blake’s pouch spontaneously disappears. However, hydrocephalus may develop postnatally in children with a persistent Blake’s pouch. In the case of additional structural (CNS and extra-CNS) or genetic abnormalities, the incidence of neurodevelopmental disabilities has been described in 20% of the cases, and genetic testing should always be offered [64]. Trisomy 21 has been described in association with Blake’s pouch, even in the absence of other malformations [34].

#### 3.3.2. Inferior Vermian Hypoplasia

The partial absence of the inferior cerebellar vermis is one of the most common cerebellar anomalies referred to in fetal counseling. In the literature, vermian anomalies have been described with different terminology, and a consensus has not been reached [65]. Even though hypoplasia refers to a normal structure that is only smaller than that which is expected for the child’s age, the term inferior vermian hypoplasia is currently used to describe the absence of the inferior portion, and this will be used in this review. Vermian anomalies must be differentiated from other entities, such as Dandy–Walker malformation, molar tooth sign, cystic compression of the posterior fossa structures. However, Blake’s pouch, vermian hypoplasia, and Dandy–Walker malformation may be a continuum of anomalies, which are difficult to differentiate [66]. Nevertheless, the term “Dandy Walker variant”, describing isolated vermian anomalies, is misleading, giving the erroneous perception of an unfavorable prognosis, and it should therefore not be used. Only a few studies have assessed the children with this condition using standardized tests [34,67]. Tarui et al. reported on a small group of children from infancy to school age, and they demonstrated normal developmental outcomes in all but two of the children [37]. The postnatal MRI of the latter cases with an abnormal neurodevelopmental outcome were found to have additional malformations which were missed by the fetal MRI [37]. Therefore, the authors recommend performing confirmatory postnatal imaging. In addition, it has been described in postnatal studies that there might be a normalization of the inferior vermian hypoplasia, suggesting that the vermian growth may still proceed after 18 weeks of gestation [68]. However, inferior vermian hypoplasia can be the only fetal malformation in syndromic conditions, and accurate fetal screening and a postnatal follow-up is recommended.

#### 3.3.3. Dandy–Walker Malformation (DWM)

DWM is a very rare entity (6.79 per 100,000 births), and it is still controversial in terms of its definition and prognosis [69,70]. The Blake’s pouch, retrocerebellar arachnoid cyst, mega cisterna magna, and abnormalities of the vermian and cerebellar morphology should be considered to be differential diagnoses. The cerebellar size in mega cisterna magna is usually preserved, while in the case of a retrocerebellar cyst and a Blake’s pouch, it might be compressed. A new definition of DWM has recently been published [71]. The posterior fossa size and torcular position have been removed from the original report. DWM is now characterized by “inferior predominant vermian hypoplasia, an enlarged tegmento-vermian angle, inferolateral displacement of the tela choroidea/choroid plexus, an obtuse fastigial recess, and an unpaired caudal lobule” [71]. Classic DWM is frequently associated with hydrocephalus, requiring postnatal intervention in over 60% of cases [34]. In addition, supratentorial and extra-CNS malformations are frequently described. Although it is considered to be a sporadic condition, several monogenic and chromosomal abnormalities have been described especially in non-isolated forms [35]. In the latter, the neurodevelopmental outcome is unfavorable. On the other hand, in the absence of other malformations, more than 40% of children may have a normal development [34]. The vermian dimension and preserved lobulation seem to be an important prognostic factor for a good neurodevelopmental outcome, and they might be a valuable feature for fetal counseling [72]. However, in most of the studies, the neurodevelopmental outcome is not assessed with formal and standardized tests [67]. More recently, the quantitative analysis of fetal MRI showed that fetuses with DWM had a larger cortical plate volume compared to the control fetuses, with subtle alterations in their brain development [73]. In this study, it has been suggested that the fetuses with vermian hypoplasia have subtle cerebral volumetric abnormalities, whereas the fetuses with normal vermis have normal supratentorial volumes [73]. The authors hypothesized that brain abnormalities may contribute to the vast range of disabilities found in DWM which cannot be explained only by cerebellar malformations [73].

#### 3.3.4. Mega Cisterna Magna (MCM)

The anteroposterior diameter of the cisterna magna remains stable (2–10 mm) after 20 weeks of gestation [10]. MCM is a cisterna magna larger than 10 mm, and it is considered to be a common finding. A normal vermis appearance and the absence of communication with the fourth ventricle distinguish MCM from other conditions. It has been suggested by several authors that it may be a variant of the norm, especially in male fetuses [74]. However, it has been anecdotally reported in association with psychiatric disorders [75]. It has been rarely described in association with genetic abnormalities (e.g., trisomy 18, 13, and Turner syndrome) or additional CNS and extra-CNS anomalies. A meta-analysis reported a risk of unfavorable outcomes in 13.8% of the cases [34]. More specifically, this meta-analysis includes a case–control study, in which it reported a higher-than-expected risk of developmental disorders in this population (15%) which were unrelated to the size of the cisterna magna [28]. In the latter study, four children presented borderline developmental quotients (developmental quotient: 70–85). A detailed description with additional neurodevelopmental impairments was provided by the authors for each child. Two of those presented mild neurodevelopmental signs: poor expressive/receptive language skills and a general neurodevelopmental delay, respectively. The remaining two children had more severe outcomes: autism spectrum disorders and suspected Joubert syndrome, respectively. The latter child presented delayed developmental milestones, dysmorphic features, ataxia, and language/attention disorders, but the parents refused further investigations to confirm the suspect of Joubert syndrome [28]. However, such a high risk of developmental disorders in this study might have been related to the inclusion of seven infants with additional malformations detected by MRI [28].

#### 3.3.5. Cerebellar Hypoplasia

Congenital cerebellar anomalies may be generated by either developmental or acquired conditions. Typically, developmental conditions may result in malformations, and are they are inherited or secondary to an alteration during organogenesis. On the other hand, acquired disruptions are defined as congenital morphologic anomalies due to the damage of a cerebellum that had a normal developmental potential. More specifically, cerebellar injuries identified in fetal life are usually hemorrhagic in origin [76,77]. Fetal cerebellar disruptions may be difficult to discriminate from genetic malformations, however, they should be considered to be two separate entities in terms of their prognosis. Cerebellar hypoplasia of a genetic origin has a high risk of developmental disabilities, including motor and cognitive impairments [78]. Massoud et al. reported the neurodevelopmental outcome at 3 years of age of twenty-three children with prenatal cerebellar hypoplasia [33]. Of them, 69% of them had a normal outcome, while 13% and 17% of them had a severe or moderate/mild impairment, respectively. The sparing of the vermis seems to carry a better prognosis [33,77]. However, the study included not only cases with isolated cerebellar hypoplasia, but also subjects with complex conditions, including genetic syndromes [33]. More recently, a small cohort of children with isolated cerebellar disruption of hemorrhagic origin has been described [32]. The neurodevelopmental outcome was assessed with standardized tests at a median age of 3 years and 6 months. All of the children had the ability to walk independently. Only one child had severe impairment, with them being affected with autism spectrum disorder. The remaining nine children had either normal development or mild/moderate impairments, including mild dyspraxia, a visuo-perceptual deficit, and a speech disorder. In this cohort of children, the vermis involvement was not strongly associated with an unfavorable outcome. Additionally, the extent of the cerebellar loss and the contralateral pons atrophy described on the postnatal MRI did not seem to suggest that there would be a poorer prognosis [32].

The underlying etiology of the acquired cerebellar disruptions is frequently missed. However, trauma, infections, fetal transfusion, medications, maternal thrombophilic disorders, and rare genetic defects of vasculogenesis or collagen have been described [79].

## 4. Postnatal Follow-Up

Antenatal counseling is just the beginning of a long journey that will continue as the child ages. Depending on the severity of the condition or the need for intensive care in the neonatal period, the delivery must be planned in advance and in the appropriate setting. Whenever possible, the first neurological examination should be scheduled in the first month of life. The neurologist will recommend additional consultations when they are appropriate. A postnatal MRI is mandatory for most of the conditions to confirm the fetal findings or exclude additional abnormalities. A neonatal MRI should be performed without sedation when the child is fed and swaddled [80]. In our experience, infants who are less than 2 months old should be submitted to MRI without sedation in order to limit the side effects and avoid a prolonged post-exam observation [81,82]. It has been reported that neonatal sedation might affect the GABAergic systems, inducing learning and behavioral disabilities later in life. In the first two months of life, an infant during a period of quiet sleep after feeding makes very few movements, and therefore, good quality MRIs can be performed. After that age, sleep movements are much more disturbing, causing MRI artefacts. Moreover, brain ultrasound monitoring is necessary in the cases of Blake’s pouch, Dandy–Walker malformation, aqueductal stenosis, intraventricular hemorrhage, and agenesis of corpus callosum associated with interhemispheric lipoma. Hydrocephalus may arise from these conditions, and serial imaging is required.

Genetic testing in fetal life may be inconclusive, and further investigations are frequently necessary, especially in corpus callosum agenesis, posterior fossa and cortical malformations. It is not infrequent to postnatally detect subtle dysmorphic features which require a correct interpretation and comparison with familiar traits. In addition, comorbidities are not uncommon in genetic syndromes; for this reason, auditory and ophthalmological examinations are often prescribed. Infants with cortical malformations should be submitted to video-polygraphic electroencephalography as soon as possible after their birth in order to detect background and/or epileptic abnormalities which may require dedicated monitoring and treatment. Neurological examinations are then scheduled at least every six months in the first year of life to detect gross motor and perceptual disabilities. Early rehabilitation intervention should be started as soon as possible in case of a suspected impairment to enhance the neuroplasticity [83].

### Neurodevelopmental Assessment

It is necessary to clearly define the outcomes during the postnatal follow-up. The neurodevelopmental outcomes may be classified as severe, moderate, or mild. Children with a ‘severe’ outcome are those with motor deficits impairing their ability to walk (cerebral palsy grade 3–5 according to the Gross Motor Classification System for cerebral palsy), a development quotient below 70, a severe behavioral disorder (autism), and a bilateral sensorineural deficit (bilateral deafness or blindness). The patients with ‘moderate’ disabilities are those with motor deficits which do not impair their ability to walk (cerebral palsy grade 2 according to the Gross Motor Classification System for cerebral palsy), a development quotient between 70–84, behavioral disorders (attention deficit and/or hyperactivity), or a unilateral sensorineural deficit. Children classified as ‘mild’ are those with minor motor/coordination deficits (dyspraxia), a transient motor delay or transient muscle tonus abnormalities, or an isolated language impairment [27]. In fetal medicine, a postnatal follow-up is often determined with telephone interviews and medical records. However, these cannot be considered to be reliable instruments in assessing the specific domains of neurodevelopment. The assessment should be performed using age-specific tools. Bayley scales III and Griffiths III are frequently used in the first three years of life. Afterwards, children’s cognitive functions may be assessed with WPPSI-III/IV (the Wechsler Preschool and Primary Scale of Intelligence (4–6 years of age)), and WISC-IV/V (the Wechsler Intelligence Scale for Children (7–16 years of age)), depending on the age of the child.

In the literature, there are a few long-term studies of children with CNS anomalies which had been detected in fetal life. A long-term follow-up is often limited to the second/third year of life. However, the gold standard would be to assess the children at school age and in adolescence. The brain undergoes significant changes, and remodeling may continue into young adulthood. The neurodevelopmental profile at three years cannot accurately predict the school age and adolescence higher brain functions. The beginning of school age (six/seven years old) may be considered the first milestone for a comprehensive assessment of their cognitive and learning abilities. However, long-term evaluations should focus not only on intellectual disability, but also on subtle neuropsychological deficits. Even in the absence of a cognitive delay, minor neurodevelopmental difficulties (attention deficit disorders, behavioral problems, learning disabilities, and psychiatric disorders) may have a significant impact on their academic and social achievements. A vast range of specific neuropsychological tests and parent/teacher questionnaires are available to assess their executive functions, behavior, social functioning, and verbal skills. In Table 2, a proposal for a structured postnatal follow-up is summarized.

## 5. Conclusions

Fetal neurology is increasingly recognized as a new subspecialty that requires specific expertise, communication skills, and team-working abilities. Assessing the prognosis of the neurological conditions in fetal life is extremely challenging, and it requires extreme caution. Fetal counseling must rely on postnatal studies that are often based on not homogeneous cohorts of children. Clinical reports should focus on groups of subjects with truly isolated malformations. For this reason, multicenter studies are needed in order to obtain larger cohorts and more statistically significant results. Nevertheless, uncovering the underlying etiology of a fetal condition may require a long time even in postnatal life, or it may never be achieved. The pediatric neurologist is then expected to continue the care of these children during postnatal life with a prolonged follow-up. The neurodevelopmental assessment should be performed with standardized tests, and telephone interviews should be avoided. Rehabilitation and specific interventions should be started as early as possible in order to optimize the results [83]. Whenever possible, the follow-up would benefit from additional long-term observations at least until adolescence in order to detect the impairments that cannot be identified early in life and offer adequate support. Mild and moderate disabilities, although considered “minor” and less disabling, can be stressful for the families involved. In cases with severe outcomes, the child neurologist plays an essential role in coordinating all of the necessary interventions. The transition from the pediatric to the adult care systems is also a very delicate process in which the child neurologist must offer extensive support.

In conclusion, the specialists of the multidisciplinary team are involved in prenatal counseling depending on their area of expertise and the specific fetal condition. Obstetricians play a critical role in fetal life. They perform dedicated neurosonography, which is essential to assess fetal brain morphology and extra-CNS abnormalities. Obstetricians can also provide assistance and guidance in the fetal MRI evaluation. When there is a condition in which it may be necessary to have postnatal intensive care or assistance, the pediatricians and neonatologists are constantly involved in antenatal counseling. They are also an essential part of the postnatal and long-term follow-up. Fetal neurological counseling cannot be limited to intrauterine life, but it is a dedicated journey throughout the lives of many families and children.

## Figures and Tables

**Table 1:** Selected fetal CNS malformations: neurodevelopmental outcome [27,28,29,30,31,32,33,34,35,36,37].

Fetal Malformation	Neurodevelopmental Outcome			Postnatal Investigations	Frequent Associated Abnormalities
	normal	Mild/moderate	severe		
Isolated ventriculomegaly	73–93%	6.9%	7.9%	Brain ultrasound, MRI ^1^	Cortical malformations, periventricular heterotopia
Isolated corpus callosum agenesis	61–70%	14–27%	8.2–12.5%	Genetic consultation, WES, MRI	Cortical malformations, midline defects, extra-CNS abnormalities
Posterior Fossa malformations					
Blake’s pouch	95–100%	0–0.5%		Brain ultrasound	Ventriculomegaly
Inferior vermian hypoplasia	85–100%	10%	5%	MRI	Cortical malformations
Dandy–Walker malformation	40%	6.4%	58.2%	MRI, genetic consultation, WES, serial brain ultrasound	Hydrocephalus, cortical malformations, corpus callosum abnormalities
Mega cisterna magna	50–100%	13.8–15%		Brain Ultrasound	Cortical malformations, ventriculomegaly, extra-CNS abnormalities
Aquired Cerebellar hypoplasia	70–69%	20–13%	17–10%	MRI, trombophilic screening in haemorrhages	Ventriculomegaly, contralateral pons atrophy

^1^ in moderate severe ventriculomegaly.

**Table 2:** Proposed follow-up of children with CNS malformation diagnosed in uterus.

Domain	1–3 Years	Pre School Age (4–6 Years)	School Age (7–11 Years)	Adolescence (12–16 Years)
Cognitive functions	-Bayley Scales III (cognitive score) -Griffiths Scales III	-WPPSI-III/IV ^1^	-WISC IV/V ^2^	-WISC IV/V ^2^
Gross motor functions	-Bayley Scales III (gross motor score) -Griffiths Scales III (gross motor quotient)	-Movement ABC II	-Movement ABC II	-Movement ABC II
Fine motor/visuo-perceptual functions	-Bayley Scales III (fine-motor score) -Griffiths Scales III (eye-hand quotient) -Visual Motor Integration Test (VMI) *	-Visual Motor Integration Test (VMI) -WPPSI III/IV ^1^ Visual Spatial Index (VSI)	-Visual Motor Integration Test (VMI) -WISC-IV/V ^2^ Visual Spatial Index (VSI)	-Visual Motor Integration Test (VMI) -WISC-IV/V ^2^ Visual Spatial Index (VSI)
Language	-Bayley III (expressive and receptive language scores) -The MacArthur-Bates Communicative Development Inventories (MB-CDIs)	-WPPSI-III/IV ^1^ Verbal IQ/Verbal Comprehension Index and (VCI) Vocabulary Acquisition Index (VAI)	-WISC IV/V ^2^ Verbal IQ/Verbal Comprehension Index (VCI)	-WISC IV/V ^2^ Verbal IQ/Verbal Comprehension Index (VCI)
Behavior and emotional screening	-SDQ ^3^ parent questionnaire *	-SDQ ^3^ parents and teachers questionnaires ^3^	-SDQ ^3^ parents and teachers questionnaires	-SDQ ^3^ parents, teachers, and self-report questionnaires
Autism Spectrum Disorders/internalizing behaviors	-Modified Checklist for Autism in Toddlers revised (M-CHAT-R questionnaire) —CBCL ^4^ questionnaire ADOS-2 ^5^.	-CBCL ^4^ parents and teachers questionnaire— ADOS-2 ^5^	-CBCL ^4^ questionnaire-parents and teachers —ADOS-2 ^5^	-CBCL ^4^ questionnaire parents, teachers, and self-report —ADOS-2 ^5^
ADHD	-Clinical observation -CBCL ^4^	-Clinical observation -CBCL ^4^	-Conners 3 questionnaire parents and teachers	-Conners 3 questionnaire parents, teachers, and self-report

* 2 years; ^1^ WPPSI III or IV: Wechsler Preschool and Primary Scale of Intelligence; ^2^ WISC-IV or V: Wechsler Intelligence Scale for Children; ^3^ SDQ: Strength and Difficulties Questionnaire; ^4^ CBCL: Child Behaviour Checklist; ^5^ ADOS-2: Autism Diagnostic Observation Schedule.
